# Access to healthcare for children with Congenital Zika Syndrome in Brazil: perspectives of mothers and health professionals

**DOI:** 10.1093/heapol/czz059

**Published:** 2019-08-01

**Authors:** Maria S V Albuquerque, Tereza M Lyra, Ana P L Melo, Sandra A Valongueiro, Thalia V B Araújo, Camila Pimentel, Martha C N Moreira, Corina H F Mendes, Marcos Nascimento, Hannah Kuper, Loveday Penn-Kekana

**Affiliations:** 1 Department of Social Medicine, Federal University of Pernambuco, Avenida Rui Barbosa, 716 - Flamengo, Rio de Janeiro, RJ, Brazil; 2 Aggeu Magalhães Institute, FIOCRUZ/PE, Av. Professor Moraes Rego, s/n - Campus da UFPE, Cidade Universitária, Recife, PE, CEP:50.740-465, Brazil; 3 Public Health Department, Faculty of Medicine, University of Pernambuco, Avenida Rui Barbosa, 716 - Flamengo, Rio de Janeiro, RJ, Brazil; 4 Federal University of Pernambuco, Avenida Rui Barbosa, 716 - Flamengo, Rio de Janeiro, RJ, Brazil; 5 Postgraduate Programme in Public Health, Federal University of Pernambuco, Avenida Rui Barbosa, 716 - Flamengo, Rio de Janeiro, RJ, Brazil; 6 National Institute of Women, Children and Adolescents Health Fernandes Figueira/Fiocruz, Avenida Rui Barbosa, 716 - Flamengo, Rio de Janeiro, RJ, Brazil; 7 Clinical Research Department, International Centre for Evidence in Disability, London School of Hygiene & Tropical Medicine, Kepple Street, London, UK; 8 Epidemiology and Public Health, Maternal and Neonatal Health Group, London School of Hygiene & Tropical Medicine, Kepple Street, London, UK

**Keywords:** Access, health system, Zika, disability

## Abstract

The Congenital Zika Syndrome (CZS) epidemic took place in Brazil between 2015 and 2017 and led to the emergence of at least 3194 children born with CZS. We explored access to healthcare services and activities in the Unified Health Service (*Sistema Único de Saúde*: SUS) from the perspective of mothers of children with CZS and professionals in the Public Healthcare Network. We carried out a qualitative, exploratory study, using semi-structured interviews, in two Brazilian states—Pernambuco, which was the epicentre of the epidemic in Brazil, and Rio de Janeiro, where the epidemic was less intense. The mothers and health professionals reported that healthcare provision was insufficient and fragmented and there were problems with follow-up care. There was a lack of co-ordination and an absence of communication between the various specialized services and between different levels of the health system. We also noted a public–private mixture in access to healthcare services, resulting from a segmented system and related to inequality of access. High reported household expenditure is an expression of the phenomenon of underfunding of the public system. The challenges that mothers and health professionals reported exposes contradictions in the health system which, although universal, does not guarantee equitable and comprehensive care. Other gaps were revealed through the outbreak. The epidemic provided visibility regarding difficulties of access for other children with disabilities determined by other causes. It also made explicit the gender inequalities that had an impact on the lives of mothers and other female caregivers, as well as an absence of the provision of care for these groups. In the face of an epidemic, the Brazilian State reproduced old fashioned forms of action—activities related to the transmitting mosquito and to prevention with an emphasis on the individual and no action related to social determinants.

**Key Messages**

- The mothers and health professionals reported that healthcare provision was insufficient and fragmented and there were problems with follow-up care.
- Communication between specialist services and other levels of the health system were problematic.
- The challenges that mothers and health professionals reported exposes contradictions in the health system which, although universal, does not guarantee equitable and comprehensive care.
- These findings illustrate problems that many people living with disabilities and their carers face with accessing the health services that they need.

## Introduction

The Zika virus (ZIKV) epidemic was identified in Brazil in early 2015, although recent studies indicate that the virus entry dates back to 2013 (Campos *et al.*, 2018). In August 2015, a high incidence of microcephaly cases was observed in newborns, in particular in the Northeast region, with its epicentre in the state of Pernambuco. This led the Brazilian Ministry of Health to declare a Public Health Emergency in 2015, which in 2016 was defined as an International Emergency by the World Health Organization/WHO according Diniz and Andrezzo (2017) and Lesser and Kitron (2016). Between the end of 2015 and November 2017, there were 15 298 notifications of children with severe growth and development issues suspected to be related to the Zika virus in Brazil, which is now collectively called ‘Congenital Zika Syndrome’ (CZS). Of these, 2572 cases occurred in the State of Pernambuco and 1123 in the State of Rio de Janeiro (Ministério da Saúde, 2018).

The link between the Zika epidemic and occurrence of CZS was quickly confirmed through the co-ordinated efforts of the research community and health system (Brasil *et al.*, 2016; de Araujo *et al.*, 2018). Children with CZS are now known to experience varied and severe impairments and health conditions, including cognitive impairment, epilepsy, visual problems and arthrogryposis. Consequently, they have high healthcare needs and are in frequent contact with the healthcare system. At the same time, CZS is strongly linked to living conditions, with higher vulnerability among populations from city peripheries, the poor and black populations (Silva, 2017; Souza *et al.*, 2018) groups who traditionally find it more difficult to access healthcare services.

In this context, a government response effort took place within the spheres of surveillance, healthcare and research activities. The Brazilian Unified Health Service (*Sistema Único de Saúde*: SUS) strives for universality, alongside comprehensiveness and equity as its philosophical pillars. Its structure approximates countries such as the United Kingdom, Sweden, Spain, Italy, Germany, France, Canada and Australia (Marques and Mendes, 2012). In reality, the goal of obtaining a universal, equitable and comprehensive healthcare network is yet to be attained, and gaps and inequalities persist (Paim *et al.*, 2011; Albuquerque *et al.*, 2013).

These gaps in service provision have been aggravated by the fiscal austerity policy adopted by the current Brazilian government, which has exacerbated SUS underfunding and further stimulated a predominance of the private health sector (Giovanella *et al.*, 2018). Furthermore, SUS faces serious challenges in a context of changes to the population’s demographic and epidemiological characteristics (Mendes, 2009; Paim *et al.*, 2011; Miranda *et al.*, 2017), driving the system to go beyond a healthcare model centred on disease or acute events, towards a form of healthcare guided by service integration from the perspective of continuity of care and inter-sectoral action for health promotion.

The poorest sectors of society may face the greatest difficulties in accessing healthcare through SUS (Lesser and Kitron, 2016), and this is also the group most vulnerable to CZS. Thus, in the context of 30 years of SUS, the CZS epidemic has forced a debate about the importance of social factors in access to healthcare services and activities. This article presents the findings of our study which explored carers of children with CZS experience accessing health services in two Brazilian states.

### Access and its dimensions

Universal access to quality healthcare for everyone everywhere is a key component of the Sustainable Development Goals (SDG) and a principle focus of the global health community. However there is a lack of consensus in the literature on the best way to conceptualize and operationalize access, apart from a general agreement that it is a complex and multi-dimensional concept (Travassos and Martins, 2004; McIntyre *et al.*, 2009; Levesque *et al.*, 2013). For Penchansky and Thomas (1981), access refers to the goodness of fit between the services of health providers and the needs of their clients. Authors such as Donabedian (2003, 1973), Julio (1985), Fekete (1996), Andersen (1995) define access as the population’s capacity to seek out and obtain services when necessary, easily and conveniently, going beyond service entry and availability alone. Levesque et al. (2013) goes further still and defines access as the opportunity to identify needs, seek out healthcare services, attain, obtain or use services and to have one’s needs met. Thiede and Mcintyre (2008) incorporate into the notion of access the idea of having freedom to use healthcare services, based on the notion of empowerment, which includes information as an essential prerequisite for access. This conceptualization of access incorporates a focus on the health system itself, including professional training and the preparation of teams in relation to patient diversity, addressing distinct socio-cultural characteristics. For Pinheiro and Mattos (2005), access is a social construction, determined by the socio-cultural context, which shapes health needs in terms of perceived dimensions of supply and demand.

In this article, our conceptualization of access focuses on two basic dimensions of accessibility: (1) Organizational and Socio-cultural Accessibility and (2) Geographical and Economic Accessibility (Table 1) based on Donabedian (2003, 1973) and Fekete (1996). This article analyses access to healthcare services and activities from the perspective of mothers of children with CZS and professionals from the Public Healthcare Network in the states of Pernambuco and Rio de Janeiro in Brazil, in the aftermath of the ZIKV epidemic. This is outlined in Table 1.

**Table 1:** Table demonstrating certain organizational, socio-cultural, geographical and economic aspects of accessibility

Access concept	Access dimensions	Aspects that may be related to each dimension
Access and accessibility to healthcare services and activities have similar meanings and refer to the capacity to obtain healthcare, when necessary and easily and conveniently, and go beyond service availability (Donabedian, 1973, 2003; Fekete, 1996).	*Organizational and geographical accessibility* refers to all the aspects of provision which may facilitate or hinder a person’s capacity to use healthcare services, including the way services are distributed across a given territory (Donabedian, 1973, 2003).	Suitability of technological resources and health professionals in relation to user needs: existence, or not, of services and activities in the appropriate location and at the time required, number and experience of professionals; the way services are distributed across a given territory (distance and user travel time, including travel costs); public–private composition of healthcare provision; waiting times at entrance (delays in obtaining consultations, tests and other procedures) and continuity of care: provision of follow-up care and existence of referral and counter-referral mechanisms, communication between services and between services and users.
	*Socio-cultural and economic accessibility*—from the population’s and health system’s perspective this refers to an appreciation of the phenomena that determine the search for healthcare by users and the public–private cost of obtaining treatment and other health actions (Fekete, 1996).	In relation to users, this may involve: the individual’s perception of the severity of the disease, fear of the diagnosis and interventions, beliefs and habits, credibility at healthcare services, perception of the disease’s determinants. In relation to the health system: preparation of teams given the diversity of users with distinct socio-cultural characteristics; consumption of time, energy and financial resources in seeking and obtaining healthcare, disadvantages from loss of working days, loss of work, private cost of treatment; public–private funding. Here, one may also consider the public underfunding of the health system as a structural barrier to access.

Prepared by the authors, based on Donabedian (1973, 2003) and Fekete (1996).

## Materials and methods

This is an exploratory qualitative, reflective and critical study, which, as Marshall and Rossman (2011) note, aims to construct descriptions of complex phenomena as yet unexplored in the literature, based on an analysis of particular cases. Here, the complex phenomenon being explored is an analysis of access to healthcare services from the perspective of mothers of children with CZS as well as the health professionals who interact with them and provide their care. As Minayo (2010) reminds us, the qualitative approach is aimed at analysing the meaning that subjects attribute to facts, relationships and practices, examining both these subjects’ interpretations and their practices.

The research was conducted in two contrasting Brazilian states—Pernambuco, located in the Northeast region, and Rio de Janeiro in the Southeast. The two states have distinct social and demographic characteristics and their healthcare networks are configured differently. According to data from the National Registry of Health Facilities (*Cadastro Nacional de Estabelecimentos de Saúde*: CNES), in 2018, only 19% of Rio de Janeiro’s healthcare network was linked to SUS (the state network itself and the private network linked to SUS), while in the state of Pernambuco this figure was 55%. In terms of primary healthcare coverage, in 2014 Pernambuco had reached 88% coverage (Governo de Pernambuco, 2018), while in Rio de Janeiro this was 58% (Secretaria de Estado de Saúde do Rio de Janeiro, 2016).

The Brazilian health system has in theory adhered to a primary care approach since the 1970s, although focal and selective programmes always existed alongside PHC. It was in the first decade of the 21st century, with the implementation of the National Primary Care Policy through the Family Health Strategy/ESF (Brazil, 2006) that the scope and concept of primary care became a key focus of the government. The PHC approach adopted was based on multi-professional teams, assigned to cover the health needs of a designated area. Registration and monitoring of the population of this area, mainly using community health workers, was key to this approach. The stated aim was to organize good quality care for all including referral where necessary (Giovanella and Mendonça, 2017).

Pernambuco was the epicentre of the ZIKV epidemic, while there were reported cases in Rio de Janeiro. Research subjects were mothers of children with CZS who received care within the public health system. We purposively sampled participants to identify a range of subjects, with respect to: severity of the syndrome, age of the child, age of the caregiver, ethnicity of the mother and socio-economic status. The sample was restricted to people living in the urban areas. We also recruited health professionals from a range of specialities (e.g. paediatrician, physiotherapist) who provided care as part of the two states’ responses to the epidemic. Subjects were selected according to the inclusion criteria found in Table 2.

**Table 2:** Inclusion criteria for mothers of children with CZS and health professionals from the public healthcare network in the states of Pernambuco and Rio de Janeiro, Brazil (2017)

Research subjects	Pernambuco	Rio de Janeiro
	Inclusion criteria	Inclusion criteria
Mothers	Mothers of children with CZS who participated in the case control and cohort study in Pernambuco and who, following telephone contact, agreed to participate in the research.	Mothers of children seen at the two referral hospitals in the capital.
Professionals	Professionals from the primary healthcare network or specialized in medium and high complexity care, working in the care of children with CZS and their families, mothers in particular.	Professionals working in at least one of the two specialized referral hospitals with children with CZS and their families, mothers in particular.

Twenty-one healthcare professionals and 31 mothers were interviewed (Table 3). Interviews took place in 2017—between April and November in Pernambuco and between June and October in Rio de Janeiro. Interviews were carried out by experienced qualitative researchers who were members of the research team, using interview guides. A total of three interviewers were used in Recife and four in Rio de Janeiro. In Rio interviews with mothers were carried out in a private location in the facility, whereas in Recife the interviews were carried out in mother’s homes.

**Table 3:** Showing the research subjects and interview locations in the states of Pernambuco and Rio de Janeiro, Brazil (2017)

Research subjects	State of Pernambuco		State of Rio de Janeiro	
	Health professionals	Interview location	Health professionals	Interview location
21 Health professionals	Total: 10 (A) 05 doctors: 02 obstetricians; 01 neonatal physician; 02 specialists in Family and Community Health (B) 01 nurse specialized in Family and Community Health (C) 01 psychologist, 01 physiotherapist, 01 occupational therapist from the Specialized Child Health Clinic; (D) 01 state health surveillance manager	High Complexity Care Hospital (01); Family Health Units (03); Specialized Child Health Clinic (01); State Health Department (01)	Total: 11 (A) 04 doctors: neonatal physician, obstetrician, neuro-paediatrician, ophthalmologist; (B) 01 psychologist; (C) 02 neonatal obstetric nurses; (D) 01 social worker; (E) 01 biologist; (F) 01 hospital surveillance professional; (G) 01 nursing technician.	Interviewed in the professional’s residence (01); Hospitals Specialized in High Complexity Care (02).
31 Mothers of children with CZS	16 mothers	In the mother’s residence or at a location of her choice	15 mothers	In the 02 Hospitals Specialized in High Complexity Care

A review of policy documents related to the states response to ZIKV was also carried out (Table 3). We consulted data from the CNES and from Decrees, Ordinances, Protocols and Technical Notes related to the ZIKA epidemic and the healthcare network for children with CZS.

All interviews were digitally recorded. In addition, the interviewers took notes which were shared with the research team. The interviewers came together regularly with the senior researchers to discuss key findings, difficulties and any changes needed in the interview guides. All interviews were transcribed and the transcriptions were checked by the researchers. Interviews were then anonymized and translated into English. The entire qualitative research team met in Brazil to discuss analytical approaches in February 2017 and July 2017. The interviews were independently coded in the UK and Brazil and agreement on emerging themes was then reached in a February 2018 workshop and in conversations via Skype.

The research was carried out as part of a larger study investigating the social and economic impacts of CZS in Brazil which was funded by Welcome, UKAID and an EU Horizon 2020 grant (Kuper *et al.*, 2018). The study observed all the ethical protocols recommended by the authors institutes and received ethical approval from the Research Ethics Committees of the authors’ institutions. Healthcare professionals provided written informed consent for the interviews. The mothers of children with CZS gave verbal recorded consent after an information sheet was read, and the researcher signed to verify that this had been done. These precautions were taken in case information was revealed about abortion or other behaviours considered illegal in Brazil. All transcripts were coded to guarantee subject anonymity.

## Results and discussion

The CZS epidemic in Brazil demanded the government to respond in terms of health surveillance, health services and research activities. In relation to the organization of healthcare services, the interviews and the document review clearly show that the Pernambuco and Rio de Janeiro State Health Departments sought to extend and enable access to healthcare services for children with CZS. In 2015, Pernambuco had two referral hospitals for high complexity care located in the state capital, and by 2017, Pernambuco State Health Department supported 30 centres specialized in medium complexity care for consultations, tests/diagnostic support and rehabilitation, distributed across the 12 Health Regions (Governo de Pernambuco, 2018). However, there were questions about the quality of new services—as one state health manager noted—‘We have extended […] But, I don’t think it’s very good’ (State Health Manager, Pernambuco).

The provision of care was also uneven. In Rio de Janeiro’s State Health Department, the two hospitals providing medium and high complexity paediatric care were located in the capital, and similarly, the 17 state rehabilitation centres were, for the most part (76%), distributed in capital and within Metropolitan Health Regions I and II (Governo do Estado do Rio de Janeiro, 2016). This demonstrates that the epidemic did not change one of the fundamental features of the state health system in Rio and Pernambuco, but not unique to these regions, that there is a concentration of complex services within the capital and Metropolitan Regions (Gerschman, 2010). This organizational design constitutes a significant barrier to access for many families.

Three levels of government are involved in healthcare in Brazil. The National Department, States and Municipalities, which vary considerably in size, are the major providers of healthcare. Most of the municipalities in rural and interior parts of Brazil do not have a specialized healthcare network. In response to this Brazil developed a Regionalization Policy which aimed to overcome the fragmentation of healthcare and constraints faced by small municipalities. However, it is widely acknowledged that implementation of this policy has been problematic, with key challenges being the criteria and the tools for setting up and financing the regionalized networks of comprehensive healthcare, and the negotiation process between the three spheres of government (Vargas *et al.*, 2015).

Availability of healthcare services for children with CZS is a necessary condition, but not sufficient, to guarantee access. A functioning health system also needs to have the capacity to facilitate entrance, guarantee continuity of care and co-ordinate inter-sectoral activity. Co-ordination of care is particularly important in relation to CZS, as the children have multiple and complex health needs. However, the interviews with mothers of children with CZS and with health professionals from the public healthcare networks in the two states contained numerous accounts of mothers struggling to access services for their children related to organizational and socio-cultural, and geographical and economic accessibility.

### Organizational accessibility

During the interviews with the mothers in the two states, accounts of daily battles for access recurred; ranging from receiving a diagnosis to getting the required medical treatment, rehabilitation and surgical procedures.

*I discovered that* she *had micro and then I had her. And then the struggles began. […] She has physiotherapy but it’s cut, then it starts again. And so, it goes on… […] The medication is very expensive. […] And so now she has to have hydrocephalus surgery, and they put in the shunt… now she is 1 year and 7 months old* (Mother Recife 1).

For the healthcare professionals, the CZS epidemic exposes a bottleneck in specialized care in SUS and highlighted the unmet demand for healthcare services and activities already experienced by other children and people with disabilities (Moreira *et al.*, 2018).

*[…] the public service already experiences huge unmet demand, with toxoplasmosis, with cytomegalovirus, with cerebral palsies due to other causes. […] And now it has to deal of Zika* (Neuro-paediatrician 1 – Rio de Janeiro).

The establishment of specialized referral services in the two states, did not in itself guarantee access to these services (Governo do Estado do Rio de Janeiro, 2016; Governo de Pernambuco, 2018). The responsibility for making appointments to access services often fell to the mothers, either at the hospitals or specialized centres themselves, by seeking recommendations from health professionals within their own referral units, by telephone or through their own personal network of contacts. Mothers reported large delays in receiving the care needed, given the long waiting times to obtain care via the formal mechanisms that regulate access. Such delays and difficulties in accessing services provide a solid indication that demand is greater than supply. These issues were also recognized as a problem by most of the health professionals.

*I went spontaneously, I knocked on the door early (Specialized Public Hospital). After the baby was born. […] because of the regulation, it takes a long time. […] They ordered it now, just recently, only she is already one year and three months old* (Mother, Rio de Janeiro - 2).

*They have difficulties in making bookings, difficulties in coming back* (General practitioner, Pernambuco).

*We receive children that have never had speech and language therapy, have never had physiotherapy* (Neonatal nurse, Rio de Janeiro).

In the case of children with CZS, although there was talk of extending services, it was the perception of both mothers and professionals that the provision of care remained insufficient, concentrated in the urban areas and fragmented across several services. This pattern reflects a wider picture of difficulties accessing specialized care. Authors such as Cunha (2011) and Tesser and Neto (2017) consider this uneven distribution of services to reflect the unequal and heterogeneous structure in Brazil, which is a huge challenge for the implementation of SUS. Previous research has also highlighted difficulties in accessing appointments for specialist services. In a study conducted in Pernambuco, 85% of the interviewed professionals cited the existence of specialist care centres although only half (49%) of the 3617 users interviewed reported that Primary Care professionals were always able to book appointments for specialized care (Albuquerque *et al.*, 2014).

During the interviews, mothers from both Pernambuco and Rio de Janeiro reported, and expressed indignation about, termination of the rehabilitation treatment for children with severe disabilities due to CZS, even when there was no prospect of improvements in the child’s functional status over the short term without these treatments.

*(…) most are being discharged. They say it’s because there is no corresponding treatment. And, whether or not they want to, they need to open up slots for others […] It’s absurd! Knowing that the professional is giving up on my child when they should be persisting* (Mother, Pernambuco – 5).

Some professionals from the states of Rio de Janeiro and Pernambuco expressed concerns about follow-up care, with a lack of co-ordination or communication between the different specialized services used by the same child or the different levels of the health system (primary and specialized care). In the interviews with health professionals it was clear that most of them were unaware of other referral services or pathways established by the health departments.

*[…] we know about the fragility of primary care in the municipalities […] And so we can’t find out how these children are. […] we really aren’t able to provide follow-up care* (Paediatrician, Rio de Janeiro).

*So sometimes the mother is there, she’s just started, right? But she’s in unit A, X, Y and these services don’t talk to each other, right? […] There was a mother who said: ‘Will I have to tell them all over again… do I have to?’* (Occupational Therapist, Pernambuco).

Difficulties with the referral system have also been reported in the past by researchers in Brazil. Cunha and de Sousa Campos (2011) found that professionals often receive users through referrals without dialogue with the professionals who refer them, hindering linkages with primary care or the co-ordination of care. Terraza Núñez *et al.* (2006) highlighted the need for technology and information systems (telephone, electronic mail, case meetings and agreed referral criteria) to facilitate referral pathways and feedback between professionals from different levels of the health and social care system. Our results suggest that this is still a problem.

Difficulties in access are not only limited to specialized services aimed at children with CZS, but also affect the mothers who need care. As one example, mothers in both locations reported difficulties in accessing contraception, in cases of unplanned pregnancy, as well as the incorrect use of contraception methods. It was not unusual to hear reports from professionals and mothers of new pregnancies, almost immediately following the birth of the child with CZS. One of the professionals interviewed recognized service failures.

*We have a great deal of difficulty with this issue of ‘preparing for pregnancy’ because we also really fail as a health service in having what we call pre-conception consultations, which would be for a mother who is thinking of getting pregnant, so she can organize herself* (Obstetrician, Pernambuco – 2).

One recurring issue in most of the mothers’ and some of the professionals’ accounts, from both states, was the public–private mixture of healthcare services used. The Brazilian health system has three subsectors: the public, where services are funded by the State; the private, where services are funded in various ways, through public or private funding; and the supplementary health sector, with different types of private health plans and insurance policies [approximately one quarter of the population (Instituto Brasileiro de Geografia e Estatística/IBGE, 2015), as well as fiscal subsidies]. People use services in all three subsectors, depending on ease of access or ability to pay (Paim *et al.*, 2011). Furthermore, people with health plans continue to use SUS in emergencies and/or in for complex treatments, principally for those denied by supplementary healthcare. Indisputably, this is a segmented health sector, which causes discontinuity and inequity in access to services. The following cases, one from Rio and one from Pernambuco, were typical of accounts from many mothers.

- In Recife: *‘*[For prenatal] I had a health plan. […] For the birth, when I needed it, the plan said it was not approved’ (Mother, Pernambuco—4). The mother gave birth in a public healthcare unit, after considering several services, because of her need for Paediatric Intensive Care. Neurological and orthopaedic monitoring of the child was provided in a specialized public healthcare unit, while rehabilitation therapies came from the private health plan.
- In Rio de Janeiro—‘Through the plan… I was intending to have the baby. […] then the test really showed micro and then I was referred here, [A specialized public hospital]’ (Mother, Rio de Janeiro—3).

The pattern of shifting from private to public healthcare services was common; as one physician reported: ‘[…] 30% of our patients here have come from private clinics […] they say that they can’t find there what they get here’ (Ophthalmologist, Rio de Janeiro).

The issue of SUS underfunding and the predominance of private expenditure, by direct disbursement and through payments to health plans and insurance, is well-recognized and may worsen in the coming years (Paim *et al.*, 2011). Analysing total health expenditure in 2014, the percentage of public expenditure in Brazil was less than other countries in Latin American and the Caribbean: Brazil—46%; Columbia—75% and Costa Rica—72.5% (World Bank, 2018). However, these countries also receive significant funding, service provision and service management from the private sector and demonstrate a prevalence of segmented models with differentiated access depending on an individual’s social position (Viana *et al.*, 2011). In Brazil, the funding situation has been aggravated by the new fiscal regime, instituted in 2016 through Constitutional Amendment 95, which ordered the 20-year freezing of federal spending, which impacted on health and education. It is projected that expenditure in these two sectors will fall from 4% of GDP in 2015 to 2.7% in 20 years (Rossi and Dweck, 2016).

### Socio-cultural accessibility

ZIKV is unevenly distributed throughout Brazilian society, affecting mostly the poorer and ethnic minority communities (Souza *et al.*, 2018). Despite the clear social determinants of ZIKV, the vector control in Brazil has been largely focused on making families responsible for their environment (e.g. cleaning homes), almost entirely excluding from the debate the fact that arboviruses are, above all, the result of collective shortages in public goods, in particular a lack of adequate environmental sanitation (Lowy, 2017). A common view was that the government implemented control measures that blamed the victims, rather than recognizing the socio-environmental issues related to recurrent arboviral epidemics, such as ZIKV. ‘[…] I think the protection we provide the patient is fragile. […] I think that there should be a collective protective measure, because individual protection is so small! It is very individualized, the blame, placing all the blame on the patient, or the health professional!’ (Obstetrician, Pernambuco—2). Mothers also perceived the failure of the government policies for vector control. As one mother in Pernambuco points out: ‘It is 22 years since my sister died of haemorrhagic dengue. And after 22 years I had L. because of the mosquito, right?’ Many health professionals recognized the importance of the social determinants of the ZIKV epidemic ‘[…] the issue, in my eyes, is the lack of basic sanitation’. This belief was also seen in the statements this professional heard from others—they (the mothers) say: ‘[…] my son is like this because where I live there is no sanitation. […] sewage is bubbling up in my bathroom!’ So, I believe that they (the mothers) have this awareness, you know?—‘My son could have been born normal!’ (Physiotherapist, Pernambuco). Other health professionals were less aware of the importance of social conditions in determining the disease. On describing the birth of her child, one mother emphasized the blame imputed to her by her doctor: ‘Who told you to go and live near mosquitos?’ (Mother, Pernambuco—8). The lack of understanding about the social determinants of the disease is clear in this statement, as if the choice of where to live was equal across society.

Another important concern was in the socio-cultural distance between health professionals and mothers, and the impact this had on access and quality of care. A number of the health professionals expressed the view that some mother had a limited understanding of the severity of the disease: ‘I see that the mothers have some difficulty understanding what needs to be done, even if they are linked to the service’ (General Practitioner, Pernambuco—2). However, some professionals recognized their own difficulties in communicating the diagnosis*—*‘[…] the communication was very difficult, to show that mother, that baby’ (Neonatal Physician, Pernambuco—1). Mothers also reported negative experiences at the time the diagnosis was communicated: ‘When her head was crowning, she (the doctor) was saying: one more micro! One more micro! She went out and came back with several doctors. And I asked, what is micro? What is micro?’ (Mother, Pernambuco—4).

These findings reveal the socio-cultural distance between professionals and mothers and the hegemonic unidirectional model of communication. This is an important concern given the large impact for a family of the birth of a baby with complex health needs and how important it is for them to understand the causes and consequences of the condition (Brunhara and Eucia Beatriz Lopes, 1998). Furthermore, the way information is introduced to users may be a mechanism for expanding access (Thiede and Mcintyre, 2008), by empowering and encouraging parents (or not) to engage with services. Other authors have also recognized that healthcare professionals in Brazil still do not use communicative practices which consider the user’s diverse historical and social circumstances (Coriolano-Marius *et al.*, 2014). The communication process should have as its basis the existence of welcoming and empathic attitudes between the professional and the patient, demonstrating interest for the other and clarity when transmitting information about a given event. Currently, these attitudes are not universally observed.

### Geographical and economic accessibility

Geographical and economic accessibility addresses distance to facilities, private household expenditure on medication, transport and healthcare services, the impact on the mothers’ lives and issues related to continuity of care and the difficulties beneficiaries have accessing Social Welfare and grants. These factors inter-relate.

The fragmented provision of care in a number of services, both in the urban and rural areas, is highlighted as a concern for most of the mothers from Pernambuco and some professionals from both states. Difficulties in reaching services are further aggravated by high household expenditure on transport and increased travel time. Furthermore, 2 years after the epidemic, the children have become bigger and heavier, which made using the bus more difficult. These aspects interfered in treatment accessibility.

*I take three buses to get here and then to get home* (Mother, Pernambuco—8).

*The free transport pass is insufficient […] You have to spend the whole day on the bus, in the sun and the rain, it isn’t easy. The children are getting heavier* (Mother, Pernambuco—6).

*[…] Getting to another part of the city, when there is nothing direct, so the mothers have problems attending* (General Practitioner, Pernambuco—02).

The following findings demonstrate how the epidemic reinforced historical gender inequality: it is the mothers who are the main caregivers, who spend time and energy on the direct care of their children, who lose their jobs, reinforcing the process of the feminization of care and its relationship with female impoverishment. It is, in the main, mothers who fight for of access to healthcare services and other rights, such as the Continuous Benefit Payments (*Benefício da Prestação Continuada*: BPC). These services are needed, as many mothers are not able to work while caring for their child.

*I can’t work anymore. Because I have to take care of her* (Mother, Pernambuco—9).

*And they sent me away (from work) when he reached his first birthday* (Mother, Rio de Janeiro—5).

*Hey people this mother needs help […] it’s not only the child* (Occupational Therapist—Pernambuco).

As an instrument of the social welfare systems of Brazil (Presidência da República, 1993), the BPC guarantees the monthly payment of one minimum wage to those who are elderly, 65 years and above, and people with disabilities. However, there are a series of restrictions for access such as: per capita household income below one quarter of the current minimum wage (which corresponds to US$60), as well as prohibitions on mothers from having an Employment Record Card or contributing to social security as a housewife (Silva, 2017). Several mothers and health professionals question the non-universality of the BPC and the Brazilian state’s responsibly to secure this right – ‘…] what I am most angry about, still, is being put in a position where I can’t work and I don’t have the right to her benefits […] Because they see that the person is not in a position of extreme poverty’ (Mother, Pernambuco—9). Many of the mothers interviewed argued that the benefit should not be linked to income, but rather it should be obligatory for the state to pay all families, particularly for health expenditure—‘[…] the issue of the continued benefit payment helps, but the medications are very expensive’ (Mother, Pernambuco—7). ‘The benefit does not cover everything’ (Mother, Rio de Janeiro—2). Inadequate income and poverty make access to healthcare for children with CZS more challenging, due to the high costs of transport, certain healthcare services and auxiliary treatments.

## Conclusion

The government response to the ZIKV epidemic involved strengthening of healthcare services, surveillance and research. However, most of the health professionals and mothers interviewed in this study felt that provision of healthcare for children with CZS was inadequate, fragmented, distributed across various services and concentrated in the capitals or other urban areas. Furthermore, the epidemic exposed difficulties in accessing specialized care in SUS and specifically made visible the healthcare gap into which children and people with disabilities fall.

The cross-cutting influence of poverty, as a factor that increased vulnerability to CZS and making treatment access more difficult is also clear, as well as the gender inequalities in caring. Formal support mechanisms for the families were almost non-existent. The study also identified that communication between health professionals and mothers was sometimes disrespectful and didn’t recognize the constraints that families faced. Training and awareness of health workers on communication with patients is key to ensuring that people access the service and receive good quality care from services.

The use of public and private health services reported by the mothers as a strategy reflects a pattern that has been seen in other studies. Mothers recognize that the public network presents a wider offer of care, when compared with the services offered by the health insurance plans but use private insurance to get access to certain services such as physiotherapy. Generally, most primary healthcare, and specialized care, of medium and high complexity, is carried out by the SUS. This is not only because most specialist care of high complexity is done in the public sector, but also because most private health insurance schemes have insufficient coverage especially for people which complex health needs.

High private household expenditure on healthcare illustrates the phenomenon of underfunding of the health system which increasingly incentivizes private commerce, unlike other Latin American and Caribbean countries. The case study of children with CZS exposes contradictions in the health system which, although universal, does not guarantee equitable and comprehensive care. In the face of an epidemic, the Brazilian State resorted to traditional ways of working, i.e. activities related to controlling the transmitting mosquito and to behaviour change prevention interventions which emphasized individual responsibility, and there was no action related to tackling social determinants of health. These gaps mean that the response to ZIKA was inadequate, and large gaps in healthcare access for children with CZS persist.

This response to ZIKA was to a large extent shaped by the Constitutional Amendment of 95/2016 passed by the last government, which mandated freezing federal spending for 20 years. This reflected the neoliberal position of the last government, and is supported by the current regime in Brazil. There is a concern that instead of improving the system that strove for universality, alongside comprehensiveness and equality the system is being dismantled. A key question is whether if there was another ZIKA outbreak the response would be even more inequitable and fragmented with even less of a focus on the social determinates of health.

## Ethical approval

This research was carried out as part of a larger study investigating the social and economic impacts of CZS in Brazil which was funded by Wellcome, UKAID and an EU Horizon 2020 grant (Kuper *et al.*, 2018). The study observed all the ethical protocols recommended by Brazil and England, and received ethics approved from the Research Ethics Committee of the three institutions involved (the London School of Hygiene & Tropical Medicine, the Fernandes Figueira Institute and the Aggeu Magalhães Institute, and the FIOCRUZ ethics committee). Healthcare professionals provided written informed consent for the interviews. The mothers of children with CZS gave verbal recorded consent after an information sheet was read, and the researcher signed to verify that this had been done. These precautions were taken in case information was revealed about abortion or other behaviours considered illegal in Brazil. All transcripts were coded to guarantee subject anonymity.

## References

[czz059-B1] Albuquerque M, Lima LP, Costa AM et al 2013 Regulação Assistencial no Recife: possibilidades e limites na promoção do acesso. Saúde e Sociedade 22: 223–36.

[czz059-B2] Albuquerque MSV, Lyra TM, Farias SF et al 2014 Acessibilidade aos serviços de saúde: uma análise a partir da Atenção Básica em Pernambuco. Saúde Debate 38: 182–94.

[czz059-B3] Andersen RM. 1995 Revisiting the behavioral model and access to medical care: does it matter? Journal of Health and Social Behavior 36: 1–10. 7738325

[czz059-B4] Brasil P, Pereira JP Jr, Moreira ME et al 2016 Zika virus infection in pregnant women in Rio de Janeiro. New England Journal of Medicine 375: 2321–34. 26943629 10.1056/NEJMoa1602412 PMC5323261

[czz059-B5] Brazil. 2006 Ministério da Saúde. Secretaria de Atenção á Saúde. Departamento de Atenção Básica. Política Nacional de Atenção Básica/Ministério da Saúde, Secretaria de Atenção á Saúde, Departamento de Atenção á Saúde. Brasília: Ministério da Saúde.

[czz059-B6] Brunhara FCRP, Eucia Beatriz Lopes P. 1998 Mães e filhos especiais: reações, sentimentos e explicações à deficiência da criança. Cadernos de Psicologia e Educação - Paidéia 9: 31–40.

[czz059-B7] Campos TDLDurães-Carvalho R, Rezende AM et al 2018 Revisiting key entry routes of human epidemic arboviruses into the mainland Americas through large-scale phylogenomics. International Journal of Genomics 2018: 6941735. doi: 10.1155/2018/6941735. 30402454 10.1155/2018/6941735 PMC6196792

[czz059-B8] Coriolano-Marius MWL, Manchester de Queiroga BA, Ruiz-Moreno L, Soares de Lima L. 2014 Comunicação nas práticas de saúde: revisão integrativa da literatura. Saude e Sociedade 23: 1356–69.

[czz059-B9] Cunha GT, de Sousa Campos GW. 2011 Apoio Matricial e Atenção Primária em Saúde. Saúde e Sociedade 20: 961–70.

[czz059-B10] de Araujo TVB, Ximenes RAA, Miranda-Filho DB et al 2018 Association between microcephaly, Zika virus infection, and other risk factors in Brazil: final report of a case-control study. The Lancet. Infectious Diseases 18: 328–36. 29242091 10.1016/S1473-3099(17)30727-2 PMC7617036

[czz059-B11] Diniz SG, Andrezzo HF. 2017 Zika virus—the glamour of a new illness, the practical abandonment of the mothers and new evidence on uncertain causality. Reproductive Health Matters 25: 21–5. 29227208 10.1080/09688080.2017.1397442

[czz059-B12] Donabedian A. 1973 Aspects of Medical Care Administration. Boston, MA: Harvard University Press.

[czz059-B13] Donabedian A. 2003 An Introduction to Quality Assurance in Health Care*.* New York, NY: Oxford University Press.

[czz059-B14] Fekete MC. 1996 Estudo da acessibilidade na avaliação dos serviços. Projeto GERUS. sl. sn.

[czz059-B16] Gerschman S. 2010 Formulação e implementação de políticas no Estado do Rio de Janeiro In: UGÁ D. M. A. S. C., DE M., Martins M, Neto BFC (eds) A Gestão Do SUS No Âmbito Estadual o Caso Do Rio De Janeiro. Rio de Janeiro: Editora Fiocruz, 69–88.

[czz059-B17] Giovanella L, Mendonça MHM. 2017 Atenção Primária à Saúde In: Giovanella L, Mendoza-Ruiz A, Aline de Carvalho Amand Pilar et al (eds). Políticas de Saúde No Brasil. Rio de Janeiro: Editora FIOCRUZ, 2^a^ Edição., 493–545.

[czz059-B18] Giovanella L, Mendoza-Ruiz A, Pilar A et al 2018 Sistema universal de saúde e cobertura universal: desvendando pressupostos e estratégias. Ciência & Saúde Coletiva 23: 1763–76. 29972485 10.1590/1413-81232018236.05562018

[czz059-B19] Governo de Pernambuco. 2018 *Rede de Assistência Aos Pacientes de Síndrome Congênita de Zika Vírus* In: Secretaria de Estado de Saúde (ed). Recife, 1–20.

[czz059-B20] Governo do Estado do Rio de Janeiro. 2016 *Nota Técnica Conjunta No 001 Secretaria de Estado de Assistência Social e Direitos Humanos (SEASDH-RJ) e Secretaria de Estado de Saúde (SES-RJ)* In: Secretaria de Estado de Saúde (ed). Rio de Janeiro, 1–11.

[czz059-B21] Instituto Brasileiro de Geografia e Estatística/IBGE. 2015 Pesquisa Nacional de Saúde 2013: acesso e utilização dos serviços de saúde, acidentes e violências. In: Instituto Brasileiro de Geografia e Estatística/IBGE (ed). *Brasil, Grandes Regiões e Unidades da Federacão*. Rio de Janeiro: IBGE, p. 105.

[czz059-B48] Julio F. 1985 El concepto y la medición de accesibilidad. Salud Pública de México Setiembre, 443. 4081889

[czz059-B49] Kuper H, Lyra TM, Moreira MEL. et al 2018 Social and economic impacts of congenital Zika syndrome in Brazil: Study protocol and rationale for a mixed-methods study. Wellcome Open Research 3: 127. 10.12688/wellcomeopenres.14838.1 PMC6807146 31667356

[czz059-B22] Lesser JK, Kitron U. 2016 A geografía social do Zika no Brasil. Estudos Avancados 30: 167–75.

[czz059-B23] Levesque JF, Harris MF, Russell G. 2013 Patient-centred access to health care: conceptualising access at the interface of health systems and populations. International Journal for Equity in Health 12: 18. 23496984 10.1186/1475-9276-12-18 PMC3610159

[czz059-B24] Lowy I. 2017 Leaking containers: success and failure in controlling the mosquito *Aedes aegypti* in Brazil. American Journal of Public Health 107: 517–24. 28207332 10.2105/AJPH.2017.303652 PMC5343710

[czz059-B25] Marques RM, Mendes Á. 2012 A problemática do financiamento da saúde pública brasileira: de 1985 a 2008. Economia e Sociedade 21: 345–62.

[czz059-B26] Marshall C, Rossman GB. 2011 Designing Qualitative Research*.* London, UK: Sage.

[czz059-B27] McIntyre D, Thiede M, Birch S. 2009 Access as a policy-relevant concept in low- and middle-income countries. Health Economics, Policy, and Law 4: 179–93. 10.1017/S1744133109004836 19187569

[czz059-B28] Mendes E. 2009 As Redes de Atenção à Saúde*.* Belo Horizonte: Autêntica.

[czz059-B29] Minayo M. 2010 O desafio do conhecimento: pesquisa qualitativa em saúde*.* São Paulo: Hucitec.

[czz059-B30] Ministério da Saúde. 2018 *Boletim Epidemiológico*. Brasília: Ministry of Health.

[czz059-B31] Miranda G, Mendes ACG, Silva A. 2017 O desafio da organização do Sistema Único de Saúde universal e resolutivo no pacto federativo brasileiro. Saúde e Sociedade 26: 329–35.

[czz059-B32] Moreira MCN, Nascimento M, Mendes CHF et al 2018 Emergency and permanence of the Zika virus epidemic: an agenda connecting research and policy. Cadernos de Saude Publica 34: e00075718 30133655 10.1590/0102-311X00075718

[czz059-B33] Paim J, Travassos C, Almeida C, Bahia L, Macinko J. 2011 The Brazilian health system: history, advances, and challenges. Lancet 377: 1778–97. 21561655 10.1016/S0140-6736(11)60054-8

[czz059-B34] Penchansky R, Thomas JW. 1981 The concept of access: definition and relationship to consumer satisfaction. Medical Care 19: 127–40. 7206846 10.1097/00005650-198102000-00001

[czz059-B35] Pinheiro R, Mattos RA. 2005 Construção social da demanda: direito á saúde, trabalho em equipe, participação e espaços públicos. Rio de Janeiro: Abrasco.

[czz059-B36] Presidência da República. 1993. Lei no. 8.742, de 7 de dezembro de 1993. Dispõe sobre a Organização da Assistência Social e dá outras providências., D.O.U, 08 de dezembro de 1993. Brasília.

[czz059-B37] Rossi P., Dweck E. 2016 Impactos del Nuevo Régimen Fiscal en la salud y la educación. Cadernos de Saúde Pública 32: e00194316. 27992044 10.1590/0102-311X00194316

[czz059-B38] Secretaria de Estado de Saúde Rio de Janeiro. 2016 Nota Técnica Conjunta No 001 SEASDH-RJ e SES-RJ. In: SAÚDE S. D. E. D. (ed.). Rio de Janeiro.

[czz059-B39] Silva AEA. 2017 Economia Política do Zika: relacões entre Estado e Cidadão. Revista Anthropologica 28: 223–46.

[czz059-B40] Souza WV, Albuquerque M, Vazquez E et al 2018 Microcephaly epidemic related to the Zika virus and living conditions in Recife, Northeast Brazil. BMC Public Health 18: 130. 29329574 10.1186/s12889-018-5039-z PMC5767029

[czz059-B41] Terraza Núñez R, Vargas Lorenzo I, Vázquez Navarrete ML. 2006 La coordinación entre niveles asistenciales: una sistematización de sus instrumentos y medidas. Gaceta Sanitaria 20: 485–95. 17198628 10.1157/13096516

[czz059-B42] Tesser CD, Neto PP. 2017 Atenção especializada ambulatorial no Sistema Único de Saúde: para superar um vazio. Ciência & Saúde Coletiva 22: 941–51. 28301001 10.1590/1413-81232017223.18842016

[czz059-B43] Thiede M, Mcintyre D. 2008 Information, communication and equitable access to health care: a conceptual note. Cadernos de Saúde Pública 24: 1168–73. 18461247 10.1590/s0102-311x2008000500025

[czz059-B44] Travassos C, Martins M. 2004 Uma revisão sobre os conceitos de acesso e utilização de serviços de saúde. Cadernos de Saúde Pública 20: S190–8. 15608933 10.1590/s0102-311x2004000800014

[czz059-B45] Vargas I, Mogollon-Perez AS, Unger J-P, da-Silva MRF, De Paepe P, Vazquez M-L. 2015 Regional-based integrated healthcare network policy in Brazil: from formulation to practice. Health Policy and Planning 30: 705–17. 24963157 10.1093/heapol/czu048

[czz059-B46] Viana LA, Costa Mda C, Paim JS, Vieira-da-Silva LM 2011 Social inequalities and the rise in violent deaths in Salvador, Bahia State, Brazil: 2000–2006. Cadernos de Saúde Pública 27(Suppl. 2): S298–308. 21789421 10.1590/s0102-311x2011001400016

[czz059-B47] World Bank. 2018*. World Development Indicators*.

